# Severe odontogenic infection: An emergency. Case report

**DOI:** 10.4317/jced.53308

**Published:** 2017-02-01

**Authors:** Marcelo Guzmán-Letelier, Claudia Crisosto-Jara, Camilo Diaz-Ricouz, Miguel Peñarrocha-Diago, David Peñarrocha-Oltra

**Affiliations:** 1 DDS, Maxillofacial Surgeon, Associate Professor, San Sebastián University Dental School. Valdivia, Chile Maxillofacial Surgeon, Hospital Base Valdivia, Chile; 2 DDS, Dental surgeon. Associate Professor, San Sebastián University Dental School. Valdivia, Chile; 3 DDS, Oral Surgery Collaborator, San Sebastián University Dental School. Valdivia, Chile; 4 MD, PhD, DDS, Chairman of Oral Surgery, Valencia University Medical and Dental School, Valencia, Spain; 5 PhD, DDS, Associate Professor Department of Stomatology, Valencia University Medical and Dental School. Valencia, Spain

## Abstract

Odontogenic infections (OI) are a major reason for consultation in dental practice. They affect people of all ages, and most of them respond well to current medical and surgical treatments. However, some OI can spread to vital and deep structures, overcome the host immune system - especially in diabetic, immunocompromised or weakened patients - and even prove fatal. Ludwig’s angina is a severe form of diffuse cellulitis that can have an acute onset and spread very rapidly, bilaterally affecting areas of the head and neck, and may prove life threatening. A case of severe dental infection is presented in which emphasis is placed on the importance of airway maintenance, followed by surgical decompression under adequate antibiotic coverage.

** Key words:**Ludwig’s angina, severe odontogenic infection, surgical decompression, dental infection.

## Introduction

Odontogenic infections (OI) are quite frequent, and usually can be resolved by local medical-surgical means - though in some cases they may become complicated and result in important morbidity-mortality (1). Odontogenic infections are generally secondary to pulp necrosis, periodontal disease, pericoronitis, apical lesions or complications of certain dental procedures (2).

The spread of an infection depends on the balance between the patient condition and microbial factors. The virulence of germs, along with the local and systemic conditions of the patient, determine host resistance (3,4). Systemic alterations favoring the spread of infection can be observed in situations such as HIV/AIDS disease, decompensated diabetes mellitus, immune depression, alcoholism or weakened states (1,3,4).

Ludwig’s angina is a head and neck infection characterized by rapid progression, with edema and necrosis of the soft tissues of the neck and floor of the mouth, and is associated to a high mortality rate (5). The disease involves progressive tumefaction of the soft tissues and simultaneous alteration of the sublingual, submandibular and submental spaces, with elevation and subsequent displacement of the tongue, which can eventually obstruct and collapse the respiratory tract (5,6). Before the age of antibiotics, the mortality rate in patients with Ludwig’s angina was over 50% (6). With the introduction of antibiotics and improvements in imaging and surgical techniques, the mortality rate has decreased to around 8% (6,7). However, in the past 10-15 years there has been a re-emergence of difficulties in managing and treating such cases, probably as a consequence of resistance to antibiotics caused by indiscriminate use, and progressive aging of the population associated to non-transmissible chronic disorders such as diabetes mellitus (3,4).

The location of the infectious process in the anatomical spaces of the buccofacial area determines the risk of compromising the respiratory tract and of affecting vital structures and organs (5). Flyn *et al.* recently simplified classification of the severity of OI, assigning a numerical score of 1 to 4 (mild, moderate, severe, extremely severe) to the anatomical spaces, according to the degree of impairment of the respiratory tract and/or vital structures such as the mediastinum, heart or contents of the cranial cavity (2). Increased severity of the infection and the appearance of complications prolong hospital stay, complicate surgical management, and place an increased demand upon Special Care Units (SCU). In this regard, the identification of risk factors associated to increased severity may be essential in order to establish early diagnosis and treatment (6-9).

We describe a case of severe odontogenic infection, and establish correlations between the disease and systemic risk factors such as diabetes mellitus and possible resistance to empirical antibiotic treatment.

## Case Report

A 42-year-old male consulted due to sudden, progressive and painful tumefaction in the left submandibular region during the last 48 hours. The disease history revealed type 2 diabetes treated with glibenclamide (50 mg/day), and arterial hypertension. Both conditions had not been followed-up on over the last 12 months. The patient suffered diabetic retinopathy and had been treated for lung tuberculosis. He had been initially diagnosed and treated by his dentist for symptoms of pericoronitis affecting tooth 3.8, with the prescription of oral antibiotics (amoxicillin 500 mg + clavulanic acid 125 mg 3 times a day) and oral nonsteroidal antiinflammatory drugs (ibuprofen 400 mg 3 times a day). Following limited response to the initial medical treatment, the patient decided to consult the maxillofacial surgery unit.

At consultation, the patient was found to be conscious, with asthenia, dehydration, fever (38.5ºC), dysphagia, severe trismus and submaxillary adenopathies. He also presented tachycardia and tachypnea (23 rpm) associated to inspiratory stridor, and with SatO2 93%. The patient showed marked facial asymmetry, with a painful indurated tumefaction in the left submandibular region, without clear boundaries.

**Figure 1:**
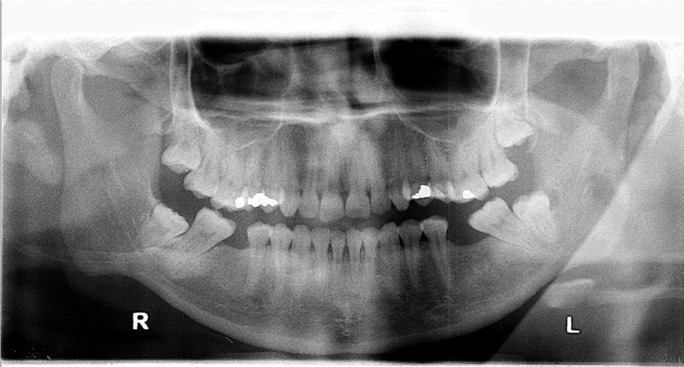
Panoramic X-ray view at initial presentation. Note the irregular pericoronal radiolucency associated to partial bony impacted tooth 3.8 in vertical position (2B Pell and Gregory). Pericoronal distal widened space compatible with paradental inflammatory cyst.

Despite the difficulty in carrying out the intraoral examination because of the trismus, a painful retromolar tumefaction was identified in relation to third molar 3.8, extending to the ipsilateral floor of the mouth. The panoramic X-ray study (Fig. 1) revealed the mentioned third molar semi-impacted in a distoangular position. A phlegmon on the floor of the mouth (Ludwig’s angina) was diagnosed, secondary to acute suppurative pericoronitis of tooth 3.8.

Due to the severity of the symptoms, the patient was hospitalized and obtained informed consent for registry and treatment physician-surgical. Empirical intravenous antibiotic therapy (clindamycin 600 mg every 8 hours and ceftriaxone 2 g every 24 hours). Upon admission the patient presented leukocytosis (20,000 cells/mm3), a C-reactive protein concentration of 300 mg/l, blood glucose 325 mg/dl and glycosylated hemoglobin (HbA1c) 17.6%. Treatment with insulin was prescribed.

**Figure 2:**
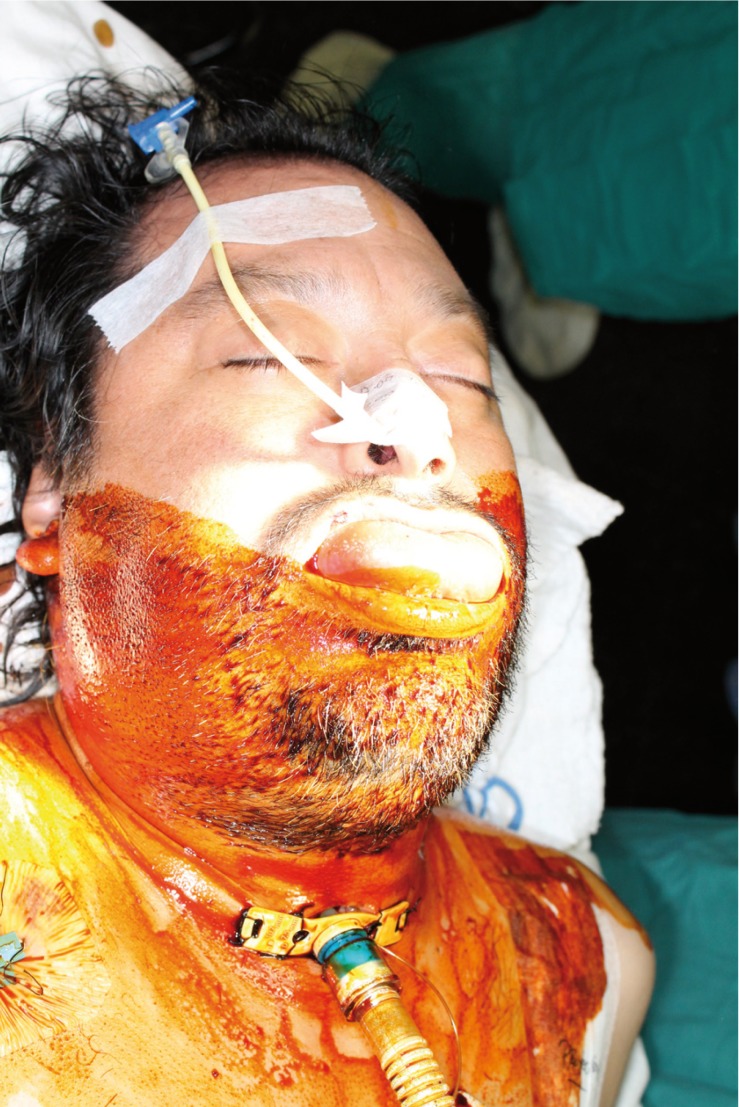
Patient with diffuse severe cellulitis (Ludwig’s angina); tracheostomy with intense swelling, simultaneous and bilateral submandibular, sublingual and submental space involvement, tongue elevation and protrusion, with total blockage of the upper airway, and protective mechanical ventilation.

**Figure 3:**
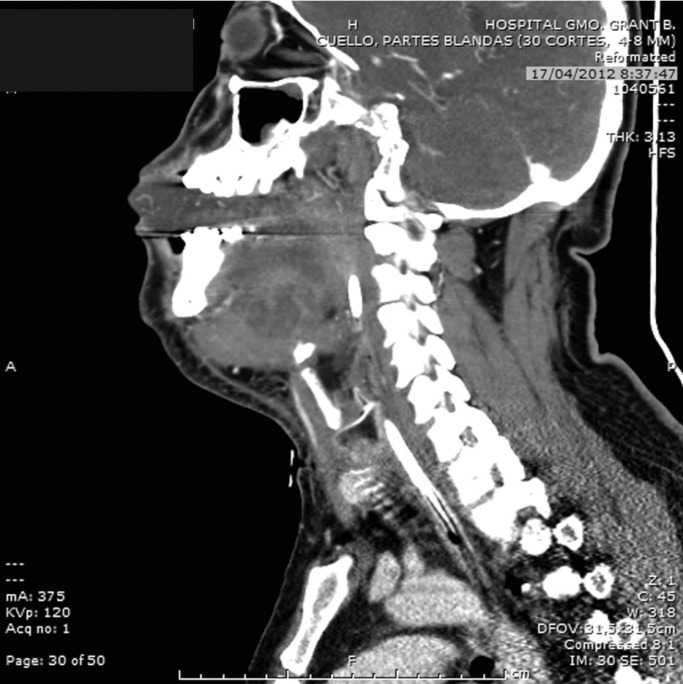
Computed tomography. Sagittal section showing upper airway elevation and protrusion of the tongue showing airway impairment and a large hypodense collection suggestive of a diffuse Ludwig’s angina infectious process.

**Figure 4:**
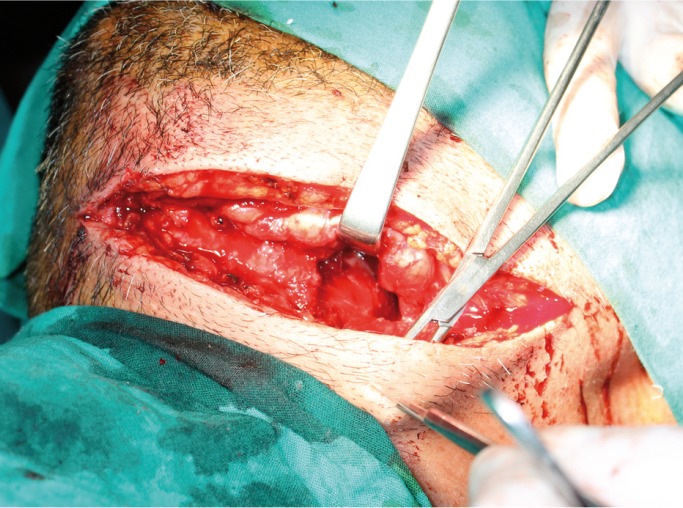
Surgical exploration cervicotomy revealing neck tissue necrosis. Debridement and necrotic debris removal was carried out, with profuse surgical irrigation.

**Figure 5:**
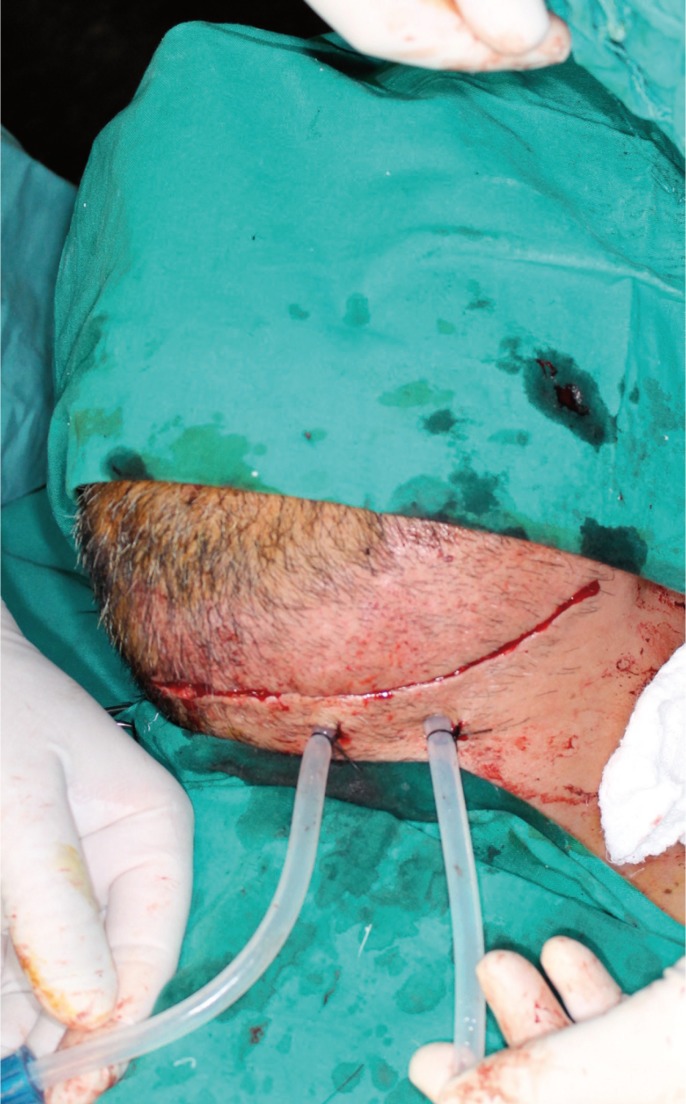
Continuous drain: two tires are placed to facilitate irrigation and aspiration of the affected tissues with purulent content.

**Figure 6:**
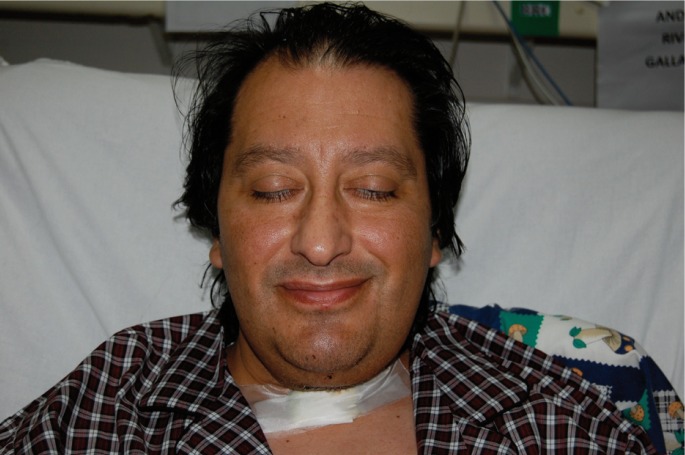
Extubated patient with removal of the tracheostomy tube. Twenty days after surgery no obvious signs of infection are noted. Marked improvement of the patient condition, with stabilization and compensation of diabetes.

Within a few hours the clinical condition worsened, with a large edema developing in the floor of the mouth and breathing difficulties. Exploration was carried out via direct laryngoscopy, and an emergency tracheotomy was performed due to the impossibility of intubation and ventilation (Fig. 2). The patient was subsequently placed under protective mechanical ventilation, and was moved to the Intensive Care Unit (ICU) for the continuation of medical management and stabilization. Following a computed tomography scan of the head and neck (Fig. 3), he developed acute renal failure with a plasma creatinine concentration of 5.7 mg/dl. On day four of admission, the causal tooth 3.8 was extracted and drained, and an extended cervicotomy was performed (Figs. 4,5). Cultures proved positive for *Acinetobacter baumannii* (AB) and methicillin-resistant *Staphylococcus aureus* (MRSA), and treatment with tigecycline was prescribed (50 mg every 12 hours i.v. during 14 days). The patient evolved favorably, with a decrease in inflammatory parameters and recovery of renal function. Extubation was carried out after two weeks, maintaining good respiratory and hemodynamic function, with a Glasgow coma score of 15 (Fig. 6). The inflammatory parameters improved, with resolution of the fever. Spontaneous ventilation was restored, without the need for additional oxygen. On day 22 of hospital admission, the patient was in good general condition, hemodynamically stable, the surgical wound showed no signs of infection, and the inflammatory parameters were found to have normalized. Discharge was therefore decided, with ambulatory checkups after 7, 14 and 30 days.

## Discussion

Ludwig’s angina was first described in 1836 by the German physician Wilhelm Friedrich Von Ludwig as a severe infectious di-sease giving rise to rapidly evolving cellulitis in the floor of the mouth. The condition is potentially serious, as it may lead to sepsis, cause obstruction of the upper airway and produce edema of the epiglottis (10,11).

The underlying infectious process may be of odontogenic or non-odontogenic origin. Odontogenic infection (OI) originates in teeth or in surrounding tissues, affecting the periapical bone from where it spreads towards either neighboring structures (continuous propagation) or structures located further away (distant propagation) (12-14). Odontogenic infections are the most frequent presentations, 70-90% originating from pulp necrosis, periodontal disease, pericoronitis, granulomas, apical cysts or complications of dental procedures. Non-odontogenic infections in turn are associated to maxillofacial fractures, submandibular sialoadenitis, infections of the salivary glands, tumor or cystic lesions, and infections of pharyngeal or tonsillar origin, among others (2,11).

The literature describes odontogenic infections as the most common cause of head and neck ailments. Umeda *et al.* (6) presented 9 cases and reviewed the English language literature, documenting 125 infections of odontogenic origin. They reported periapical infections of the second and third mandibular molars as being the most frequent origin (70-80%), due to the fact that the roots of these teeth typically extend beneath the mylohyoid muscle, producing infection that spreads into the submaxillary space, and from there to the sublingual and submental spaces, consecutively. Flynn *et al.* (2) published a study of 49 cases of severe odontogenic infections with involvement of the deep lying spaces. Of these cases, 68% were associated to inferior third molars, 22% to pericoronitis, and the rest to other mandibular posterior teeth. Our case is consistent with the origin described in the literature, involving a semi-impacted third mandibular molar with pericoronitis that evolved into a phlegmon in the floor of the mouth. Kurien *et al.* (15) carried out a comparative study of the causes of Ludwig’s angina in children and adults. They identified a dental origin in 52% of the adults, and 39% suffered predisposing systemic diseases such as poorly managed diabetes, alcohol abuse or immunosuppression.

The affected anatomical spaces of the head and neck must be identified and classified according to their potential impact upon the respiratory tract and/or vital structures such as the mediastinum, heart or cranial content. Flynn *et al.* developed a severity scale (SS) for OI in which a numerical score of 1 to 4 is respectively assigned to mild, moderate, severe and extremely severe involvement of the anatomical spaces ( Table 1). This numerical score closely relates anatomical space involvement to the risk of affecting the respiratory tract and vital structures. According to the mentioned classification, if a patient has more than one compromised space, a sum of all values is made. In our case, the patient showed involvement of the submaxillary space (SS=2), sublingual space (SS=2), submental space (SS=2), pterygoid mandibular space (SS=2) and lateral pharyngeal space (SS=3) - thus yielding a total of 11 points out of a maximum of 36 points. In our opinion, this classification alone is unable to offer a clear idea of the true severity of the infection, since the sum obtained did not even reach half of the maximum score.

**Table 1:**
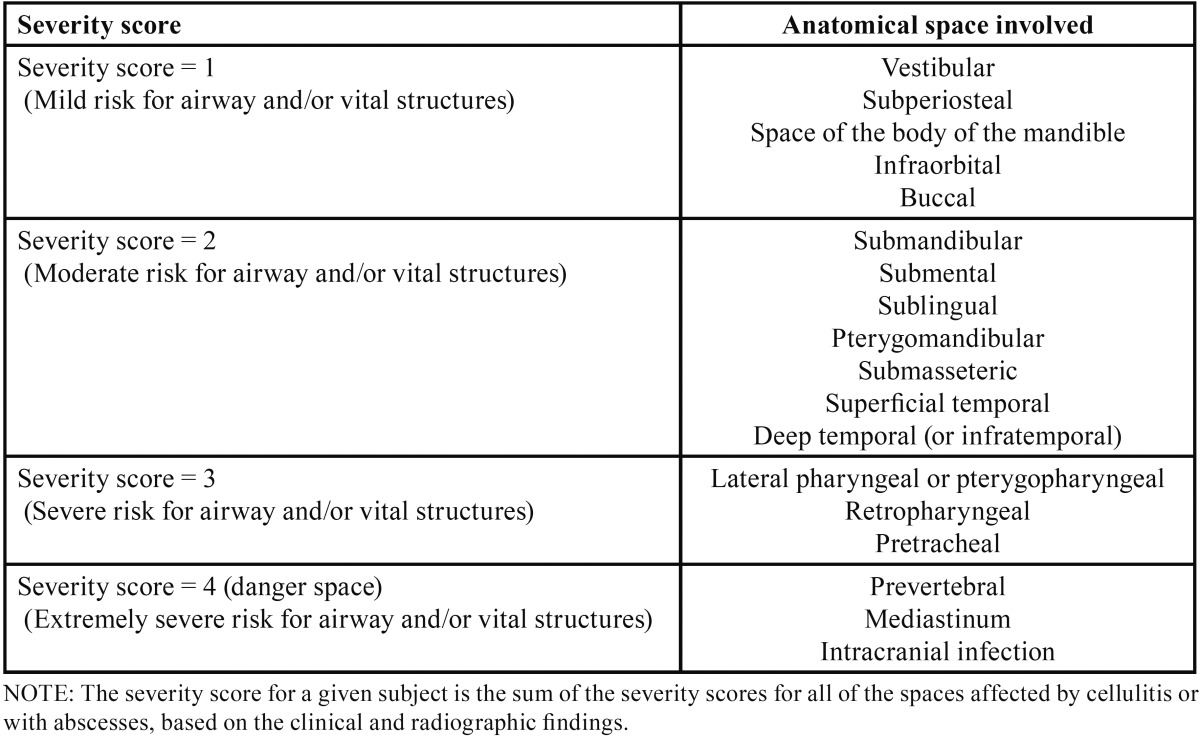
Severity scores for severe odontogenic infections according to anatomical space involvement. (Reproduced from Flynn *et al.* Severe Odontogenic Infections. J Oral Maxillofac Surg 2006).

The most frequent cause of death in patients with OI is respiratory tract obstruction (5,7). The physician therefore must evaluate this aspect at initial patient assessment. It is of great importance to identify certain signs and symptoms when anatomical spaces are compromised ( Table 1).

Trismus is an obvious sign suggestive of serious OI. Buccal opening that has diminished 20 mm or more in a short period of time, with severe pain, is considered to indicate infection of the perimandibular anatomical spaces until proven otherwise (2,8,10). Nonetheless, regardless of trismus, the attending physician must assess the presence of dysphagia and visualize the oropharynx in search of a possible infectious process.

In cases of partial obstruction of the respiratory tract, abnormal sounds will be heard, such as stridency and wheezing, due to the turbulent passing of air through the respiratory tract. In these cases the patient typically inclines the head frontward or moves the neck towards the opposite shoulder in order to straighten the respiratory tract and thus improve ventilation (10). Oxygen saturation below 94% in a previously healthy patient is a sign of insufficient oxygenation of the tissues. When accompanied by clinical signs of partial or total obstruction, it constitutes a surgical emergency, and urgent endotracheal intubation must be performed in order to secure the respiratory tract via a tracheotomy or cricothyrotomy as in our case.

In several studies (2,4,8), the initial leukocyte count has been cited as an important predictor during hospital admission. Leukocytosis above 12,000 cells/mm3 generates a systemic inflammatory response syndrome (SIRS), which is an important factor in determining hospital admission due to OI (13). In 66% of their cases, Flynn et al. were able to correlate hospital stay to the severity scale values and leukocyte counts at hospital admission. In our patient the leukocyte count upon admission was 20,000 cells/mm3, with fever (38.5ºC), causing an increase in metabolic and cardiovascular demand beyond the reserve capacity, where loss of fluids is greatly increased and entails severe dehydration.

Certain medical conditions can interfere with functions of the immune system that are essential for host defense against OI. Diabetes mellitus (DM) is the most prevalent chronic disease affecting the immune system (7,9) - hyperglycemia being the main etiological factor of DM leading to dysfunction of the immune system. All the main immune cell types are affected. In this regard, neutrophil adhesion, chemotaxis and phagocytosis are altered (10-12), and this results in a less efficient defense against microbial attack (13).

Diabetes is also characterized by exacerbated macrophage reactions, which increase the production of proinflammatory cytokines and intensify connective tissue metalloproteinase response - resulting in difficulties for containing infection (9). On the other hand, chronic hyperglycemia can exert an influence upon fibroblast proliferation and collagen synthesis, hindering tissue replacement and wound healing (9,10).

Our patient presented a glycosylated hemoglobin (HbA1c) value of 17%, consistent with the lack of disease follow-up over the last 12 months. This poor metabolic control increased patient susceptibility to immune system alterations. Hospital stay in diabetic patients with OI is much longer than in non-diabetic patients, and head and neck space involvement is also more frequent than in non-diabetic individuals. A strong link therefore exists between diabetes and complications in the management of severe OI (7-9).

The place where the patient should be operated upon (i.e., in a specialized center or on an outpatient basis) is decided from the data obtained at initial examination (7,8). In our case, the patient suffered decompensated systemic disorders such as DM, and presented clinical characteristics confirming the seriousness of OI. Accordingly, emphasis was placed on compensating the basal disease conditions before performing surgery (13).

Regardless of the severity of OI, surgical management is based on two principles: elimination of the causal focal point of infection, and surgical voiding of the compromised anatomical spaces together with adequate drainage (10-12). Surgical management of the compromised anatomical spaces must be made aggressively and promptly, as initially described by William and Guralnick (14). This approach is based on the concept that prompt emptying and surgical drainage nullifies the propagation of infection towards deeper and more severe spaces, even if the infection is in a phlegmon state (13). Samples for microbiological culture and antibiogram can be obtained at this stage. However, since the results take some time in becoming available, this practice should be reserved for cases where OI affects multiple spaces, or in patients with immune system alterations (2,7). In our patient surgery was performed on day four of hospital admission, once the basal disease conditions had been evaluated and the airway had been secured, and consisted of elimination of the causal focal point of infection and extensive cervicotomy.
